# Filmed Monologue Vignettes: a novel method for investigating how clinicians document consultations in electronic health records

**DOI:** 10.23889/ijpds.v3i1.430

**Published:** 2018-11-14

**Authors:** Simon Glew, Elizabeth M Ford, Helen Elizabeth Smith

**Affiliations:** 1 Division of Primary Care and Public Health, Brighton and Sussex Medical School, Brighton, UK; 2 Family Medicine and Primary Care, Lee Kong Chian School of Medicine, Nanyang Technical University Singapore, Singapore

## Abstract

**Introduction and Objectives:**

The accuracy of conclusions from research based on Electronic Healthcare Records (EHRs) is highly dependent on the correct selection of descriptors (codes) by users, but few methods exist for examining quality and drivers of documentation. We aimed to evaluate the feasibility and acceptability of filmed vignette monologues as a resource-light method of assessing and comparing how different EHR users record the same clinical scenario.

**Methods:**

Six short monologues portraying simulated patients presenting allergic conditions to their General Practitioners were filmed head-on then electronically distributed for the study; no researcher was present during data collection. The method was assessed by participant uptake, reported ease of completion by participants, compliance with instructions, the receipt of interpretable data by researchers, and participant perceptions of vignette quality, realism and information content.

**Results:**

Twenty-two participants completed the study, reporting only minor difficulties. 132 screenshots were returned electronically, enabling analysis of codes, free text and EHR features. Participants assigned a quality rating of 7.7/10 (range 2-10) to the vignettes and rated the extent to which vignettes reflected real-life at 93% (range 86-100%). Between 1 and 2 hours were required to complete the task. Full compliance with instructions varied between participants, but was largely successful.

**Conclusions:**

Filmed monologues are a reproducible, standardized method, which require relatively few resources, yet allow clear assessment of clinicians’ and EHRs systems’ impact on documentation. The novel nature of this method necessitates clear instructions, so participants can fully complete the study without face-to-face researcher supervision

## Background and Significance

The use of an Electronic Healthcare Record (EHR) is near universal within UK primary care. In England alone over 370 million consultations are recorded annually according to the Royal College of General Practice(1). To document a patient consultation, clinicians assign a headline descriptor, known as a Read code (2), that may be a diagnosis, symptom, sign, investigation or procedure. Read codes were created in the UK based on clinical parameters and usage. Read codes form a clinical hierarchical structured vocabulary that contains 110,000 concepts in its dictionary. Each code has a 5-byte alphanumeric code and an accompanying plain English clinical term, for example “TJ002 - Adverse reaction to Flucloxacillin”. The adjoining section of the EHR is used for narrative ‘free text’, which may include more symptoms and signs, but also impressions and reasoning, differential diagnoses, messages for other clinicians, and management plans. These records are extracted anonymously from contributing clinics and managed in large repositories such as the Clinical Practice Research Datalink (CPRD), Q Research, ResearchOne and The Health Improvement Network (THIN). These databases are used increasingly for epidemiology and drug safety research. In 2017 alone, 222 papers were published using data from the CPRD (3). However, for information governance reasons, free text portions of the record are not extracted, and currently clinical information recorded in these sections is lost to researchers.

### Data Quality

The validity of any research based on EHR repositories is dependent on the quality of the data. As EHRs contain data recorded within routine clinical care, rather than in the context of research, there are specific data quality issues of which researchers need to be mindful. One such example is in the context of case finding. In health record research, identifying cases with the entity of interest is usually achieved by the a priori compilation of a list of Read codes or the creation of an algorithm of relevant clinical information. These case-finding mechanisms favour specificity over sensitivity; consequently when a patient does not meet the criteria for the clinical entity of interest, we do not know whether they are a negative case, or a “positive, but unlabelled” case (4).

Concerns have been raised about the accuracy of coded diagnoses; the “garbage in, garbage out” concept currently has unquantifiable repercussions for the accuracy and quality of EHR database research (5, 6). Data quality is dependent on the rigour with which clinicians capture clinical information in the EHR (7), and this is influenced by multiple variables which impact on the consultation and its documentation (5, 8-12). Relatively little is known about how different clinicians document patient interactions, but there is evidence that early in the diagnostic journey, when uncertainty around a condition is greatest, clinicians favour descriptive symptom codes rather than diagnostic codes, preferring to document the differential diagnoses in the free text section of the EHR. Such an example would be the preliminary use of “cough” as a code rather than “bronchial carcinoma”, the suspected diagnosis only being captured in free text (12-14). Researchers’ case definitions usually do not include all such relevant symptom codes, thus case definitions lack sensitivity to this mode of documentation, resulting in a misinterpretation of GPs’ diagnostic awareness based on diagnostic codes alone. As a result, conclusions drawn from EHR research studies may be inaccurate, misleading (15) and, at best, require cautious interpretation (15, 16).

### Investigating data recording

Enhancing our understanding of how clinicians utilise the EHR is vital if we are to improve the rigour of EHR studies and the interpretation of their findings (5). Most previous studies have employed labour intensive methods, such as videoing consultations with actors or simulated patients to standardise the patient presentation (17), or the videoing of real consultations (18). Whilst video recording consultations are viewed as the optimal observation strategy against which the accuracy of medical record keeping can be assessed (19), real-life patient and clinician variability means patient consultations contain many uncontrollable factors. Both patient and practitioner are aware they are being recorded and, although there is some evidence this doesn’t significantly affect the consultation process (20, 21), it may introduce a selection bias in the characters of doctors and patients who volunteer to be recorded (21). To explore the impact of this potential bias, follow up video elicitation interviews can be used (post-event analysis with the participant to explore actions, beliefs and motivations) (22), but these are logistically demanding and time consuming due to the vast amounts of data generated (23, 24). In addition, filming real patients, although authentic, requires consideration of ethical, medico-legal and confidentiality issues (23, 25), as well as the organisational challenges of involving large numbers of patients to enable comparison of the EHR-documentation relating to specific conditions between different clinicians (17).

In an attempt to standardize investigation of EHR documentation, professional actors have been used as simulated patients. This reduces patient variability, whilst maintaining some degree of realism (17). However, this approach still contains a degree of inconsistency, as the information available to code is dependent on the doctor’s communication and history-taking skills, and thus assesses skills beyond the use of the EHR. Some doctor participants require time to “get into role” and pictures used to portray symptoms (such as a swollen joint) “break the spell.” Unexpected lines of questioning may also require the actors to ad lib. Logistical difficulties coordinating doctors, actors and technicians and finding space to undertake the research around busy schedules are further challenges.

In summary, the videoing of consultations with real patients, or with actors, does not allow exploration of documentation in a fully standardized and reproducible manner, and both are demanding of time and resources. We therefore sought to develop a pragmatic and low-cost alternative for exploring clinician recording in the EHR of the content of consultations. We developed filmed monologue vignettes (FMV) that could be viewed in the clinicians’ own environment, using their own computer and EHR, at a time that is convenient to them. Films allow the study to be revisited using exactly the same patient and explored across a geographically diverse area unobtrusively and requiring fewer resources than other methods. The data generated could be electronically transmitted for central analysis. This would enable effective sampling of multiple clinicians and EHRs to further understanding of, and compare, how clinicians document in their EHR system.

### Study Aims

This study aimed to pilot and test a new method of studying clinician–record interaction, by presenting clinicians with filmed monologue vignettes. The technique was evaluated for:

1. The ability to collect and generate analysable data, assessed by participant uptake, reported ease of completion by participants, compliance with instructions, and the receipt of interpretable data by researchers.
2. Participant perceptions of vignette quality, realism and information content.

The results of the coding data generated will be presented in a separate paper.

## Materials and Methods

### Ethics

The study did not require National Research Ethics Service review, as participants were NHS employees recruited solely by virtue of their professional role.(26) Approvals were obtained from the local dissertation panel (similar to an internal review board) and the medical school research governance and ethics committee, with sponsorship provided by the latter.

### Filmed Monologue Vignettes - content

Vignettes were written by two General Practitioner clinicians. They were designed to be short (less than one minute) but to contain enough information to allow a descriptor to be assigned. The content focused on allergy-related conditions, as these include problems of high clinical importance affecting multiple physical systems and all ages and can require the use of special recording features within the EHR. The vignettes were intended to reflect real-life so some included subtle uncertainties associated with diagnosis. Investigation of participants’ clinical knowledge and prescribing habits was not the aim.

### Filmed Monologue Vignettes - filming

**Figure 1: Vignette stills including their clinical presentation:**
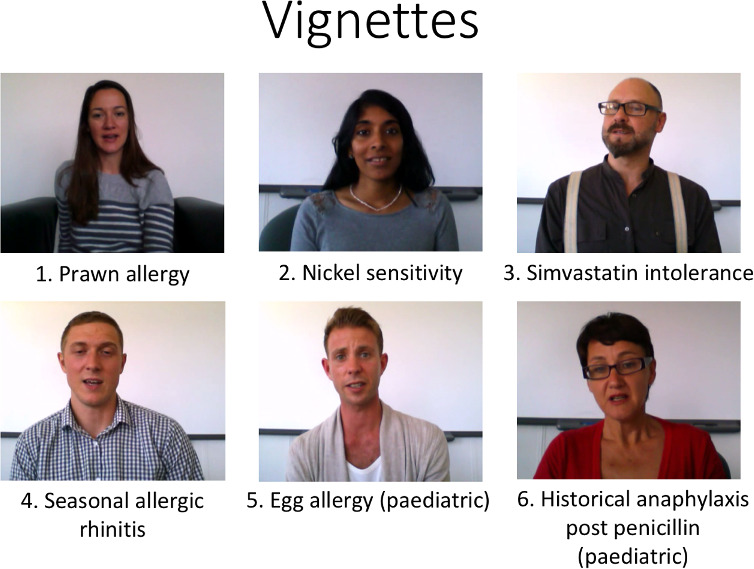


Six colleagues with no/limited acting experience were recruited. Following a brief period of preparation and direction from the researchers, they were filmed reading the vignette scripts in a head-on orientation (Figure 1) as if they were patients presenting for a consultation with the participant. Two actors portrayed parents describing their children’s symptoms to enable inclusion of paediatric conditions. The vignettes were edited into a single film using “VideoSpiritPro” v1.9 (27). Still images of skin rashes were edited into two vignettes. Each filmed vignette lasted between 21 and 50 seconds. The vignettes were edited into a single film which was 7 minutes 46 seconds in total, including a 3 minute 51 second instructional introduction. Files were available as .mpg (185Mb) and .avi (36Mb). Written instructions were also provided.

### Assessment of method:

#### 1) Questionnaire

A questionnaire was designed to evaluate the quality, realism and content of the vignettes, and the participant experience of choosing codes/text. It additionally gathered participant demographics, and explored confidence and experience with technology, the EHR and managing allergy (full questionnaire in Supplementary File 1). After each vignette, participants answered the questions in Table 1 to assess the filmed vignette monologue process.

**Table 1: Questions used to assess the quality of vignettes and their answer modalities:** 

Question	Response	Follow on

“Were you satisfied that the Read code you assigned captured the problem accurately?”	Yes/No	“If no, was it because (check box):” “Read code does not exist? Multiple Read codes are suitable? Unable to find suitable Read code? Other? (Please describe).” (Free text response)
“How important do you think the free text information is in recording this scenario?”	10cm visual analogue scale from “Not at all important” to “Extremely important”	“Please explain your response” (Free text response)
“Did you have enough information to record the consultation effectively?”	Yes/No	“If no, what additional information would you have required to record this effectively?” (Free text response)
“Rate the quality of the vignette”	1= poor, 5 = average, 10 = excellent	
“Do you feel the vignette reflected real-life?”	Yes/No	

#### 2) Evaluation of feasibility

The technique was evaluated for its feasibility to collect and generate analysable data. This was assessed according to participant uptake, reported ease of completion by participants, compliance with instructions, and the receipt of interpretable data by researchers.

### Procedure

#### 1) Recruitment

General Practice trainees across Kent, Surrey and Sussex Deanery region were targeted through emails, posters and oral presentations. They were encouraged to invite their trainers (qualified GPs) to participate. Qualified GPs were also targeted through oral presentation at educational meetings. No direct precedent existed to inform a power calculation for sample size. A similar study, the ALFA toolkit (18), enrolled 4 participants with a total of 22 consultations. A target of 30 participants in a 1:1 ratio of GP trainees to GPs was set in order to ensure a variety of different EHRs and GP experience were sampled. Participation was incentivised with three modest raffle prizes, but largely relied upon goodwill.

#### 2) File transfer

**Figure 2: Study Procedure:**
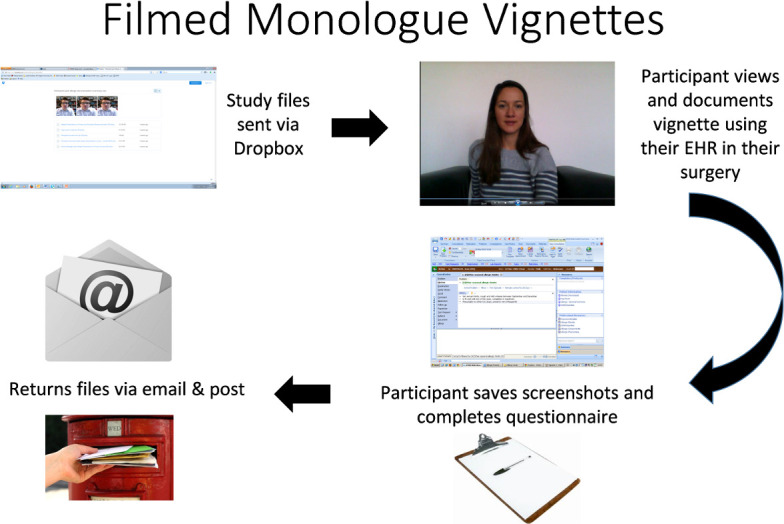


All files were transferred electronically between researcher and participant using Dropbox and secure email, apart from the return of the questionnaire. As this included Visual Analogue Scales, it could only be completed in hard copy and was therefore returned by post (Figure 2). The file pack included the questionnaire and vignette film (in 3 different file formats), written instructions of how to take a screenshot and a video file explaining how to perform the study (video transcript included as Supplementary File 2).

#### 3) Participant response to vignette

Participants were required to document the vignettes in a single, blank “dummy” patient record. The use of this new, blank, record ensured no patient identifiable information was transferred during the study. A letter to Practice Managers describing the study and requesting a blank record be created was included in the file pack. Participants were asked to document the presentation only; management plans and prescribing medication were not required.

Participants saved a screenshot of their input after documenting each vignette and, once all vignettes were completed, screenshots of the medical and allergy summary screens. A standard blank referral letter was also requested which could also be captured by screenshot and pasted into either word processing or photographic software. This was included in the study in order to identify what information was automatically transferred to referral letters by individual users and systems after data entry.

The questionnaire was printed by participants and completed by hand whilst they conducted the study, in order to avoid distracting from the EHR and video vignettes and allow completion of Visual Analogue Scales.

It was estimated that an hour would be required to complete the study.

### Data analysis

Screenshot data was anonymised by removal of any clinician identifiers prior to analysis. To ensure the technique could generate analysable data, descriptors (codes) were identified and extracted from the screen prints and, identified using NHS Read browser dictionary software (28), which also provided corresponding Read codes and hierarchical level. This ensured all valid descriptors were accounted for.

Summary screen prints and blank referral letters were analysed to see which descriptors (if any) were transferred and for relationships between the summary screen and the data on referral letters. Free text was analysed for number of words used and content. Table 2 lists the documentation aspects that are analysable using this method.

**Table 2: Analysable elements from screenshots:** 

Code	Usage, variety and frequency
	Level of code in hierarchy (inference of diagnostic certainty)

Free text	Reaction and diagnostic certainty, severity, symptoms or their absence, aetiology, differential diagnoses
	Augmentation or negation of a preceding code (not/likely/suspected)
	Number and range of words (inter-participant variability)

EHR	Features, differences

## Results

### Participants

Twenty-two participants (7 GPs and 15 GP trainees) using 4 different EHRs (SystmOne (6), EmisLV (2), EmisWeb (6) and Vision (8)) were recruited from the Kent, Surrey, Sussex deanery between January and July 2014.

### Evaluation of feasibility

The study was completed successfully with all participants geographically remote to the research team, with few technical problems reported. No difficulties were reported creating blank patient records although one participant documented in a previously used “dummy” record. Another required telephone support to download the vignette video file. One participant could not view the videos so completed the study using audio alone. One participant reported a code they had entered was not visible on their screenshot (using EMIS LV). Of the additional files requested for the study, 13 participants returned summary screens after recording all six vignettes, three participants returned blank referral letters, two participants documented their questionnaire responses electronically. Seven participants documented management plans and two prescribed medications in at least one of their vignette responses. Technical issues are summarised in Table 3.

**Table 3: Technical Feasibility:** 

Technical problems encountered	Non-blank patient record used (1)
	Telephone support required to download video file (1)
	Unable to view video (used audio alone to complete study) (1)
	Participant reported a code they had entered was not visible on their screenshot (1)

Time taken to complete study	1-2 hours

Screenshots returned after individual vignette entry	100%

Summary screenshot returned	13 (out of 22)

Blank referral letters returned	3 (out of 22)

Other deviations from instructions	Questionnaire completed electronically (2)
	Management plans created (7)
	Medications prescribed in any scenario (2)

### Assessment of vignette quality

The overall mean quality rating for all vignettes was 7.7 out of 10. Vignettes 2 (nickel sensitivity) and 4 (seasonal allergic rhinitis) rated the lowest mean scores with the widest range (Vignette 2, M= 7.1, (2-10) and Vignette 4, M = 7.2, (4-10)). Vignette 6 (historical anaphylaxis post penicillin) was rated highest (M= 8.3, (7-10)). Participants reported frustration with the low definition of the digital photograph that was used to illustrate the rash in vignette 2:

**GP4Vig2:** *“More history and examination related to necklace (needed). Couldn’t see clearly on photo.”*

### Assessment of vignette reflecting real-life

Overall, 93% of participants agreed the vignettes reflected real life. Vignettes 3 (Simvastatin intolerance) and 5 (egg allergy) were unanimously felt to reflect real life. Vignettes 2 (nickel allergy-86%) and 4 (seasonal allergic rhinitis-86%) were rated lowest.

Just over half the participants felt there was enough information in the vignettes to record the consultations effectively although this varied between vignettes (Table 4).

**Table 4: Number of codes used for each vignette, participants rating of vignette information, reflection of real-life and quality and selected comments. Version 2 and 3 refer to Read code version which is dependent on EHR used.** 

	Number of different codes used Version 2 (Version 3)	“There was enough information to record the consultation effectively” (% agree)	“This vignette reflected real-life” (% agree)	Vignette quality out of 10 Mean (range)

Vignette 1	8 (4)	55	91	7.6 (5-9)
**GP7Vig1:**“*Any breathing problems? Faintness? Worsening severity of reaction each occasion?*”				
Vignette 2	8 (6)	44	86	7.1 (2-10)
**T2Vig2:** “*Need more history. Past medical history, what creams have tried etc.*”				
Vignette 3	9 (5)	41	100	7.9 (5-10)
**T6Vig3:** “*Can only code drug reaction: free text required to document symptoms”*				
Vignette 4	9 (5)	41	86	7.2 (4-10)
**GP5Vig4:** “*This problem sounds like a viral cough (or ?smoking) I don’t want to medicalize it by adding a diagnostic label.”*				
Vignette 5	9 (7)	68	100	7.8 (7-10)
**T7Vig5:** “*Allergy is a misused term, therefore the free text is important for other health care professionals whether allergy or intolerance”*				
Vignette 6	6 (4)	68	95	8.3 (7-10)
**T7Vig6:** “*Free text needs to describe and justify code of ‘anaphylaxis’ and ‘true’ Penicillin allergy”*				
**Mean**	**8.2 (5.2)**	**53**	**93**	**7.7**
**Range**	**6-9 (4-7)**	**41-68**	**86-100**	**2-10**

### Quality of data returned for analysis

Codes were predominantly presented at the start of each entry. Occasionally, and mainly in SystmOne, additional codes/descriptors were identified within the free text, as shown in Figure 3. The NHS Read browser software (28) was successful in ascertaining each descriptor’s code and place in the Read hierarchy.

**Figure 3: Example screen prints from SystmOne (top) and Vision (bottom). 1 = Read code entry, 2 = Free text entry, 3 = Allergy entry feature, 4 = Combined Read code and free text, 5 = EHR features (patient and professional information resources):**
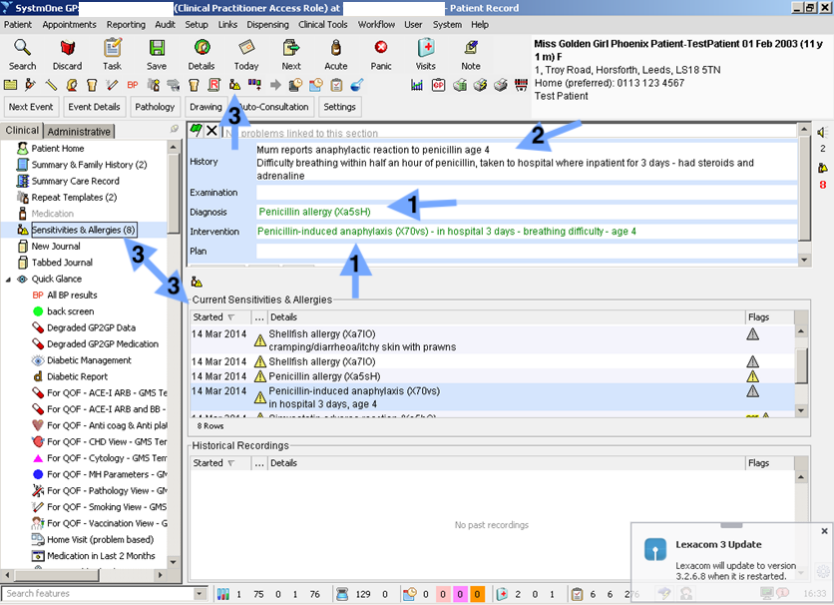

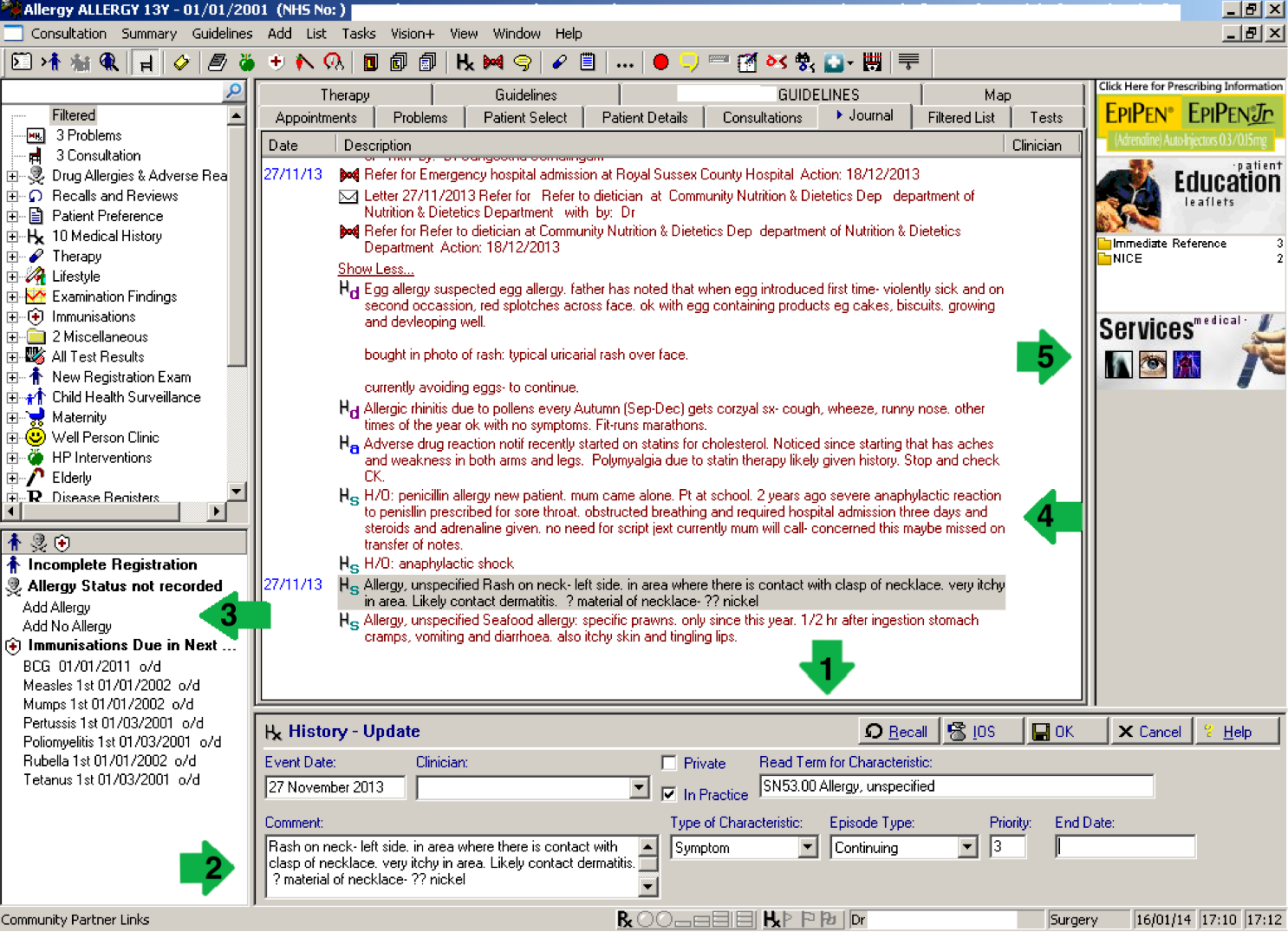


## Discussion

### Summary of findings

We have presented a novel, standardized, resource-light method for exploring how clinicians document in their own EHRs, enabling comparison of coding and free text entry. Assessment of quality and feasibility found the new method to produce good quality results and to be acceptable to clinicians. The remote nature of the study, utilizing freely available, commonly used software proved successful and provides a number of advantages to researcher and participant over previous methods of human-EHR interface research.

All vignettes were documented and returned by the 22 participants across three English counties, at various stages of their General Practice careers, sampling four different EHR systems and generating 132 consultation entries. Only minor technical issues were identified. The large amount of data generated was amenable to remote analysis by researchers. These were methodically analysed for clinical codes and content. The receipt of screen captures accurately collates inputted data as the clinician intends it to be recorded and, with enough participants, allows comparison of any impact due to different EHRs.

This method represents time and resource savings compared to conventional methods using patients or actors. Participants reported being able to fit participation around busy clinical schedules. It can be used quickly and efficiently by researchers to gain a better understanding of how the conditions of interest are recorded on the EHR and thus develop case-finding methods which match more closely GPs’ documentation methods. There is potential to reduce the resources required still further as the technology for online surveys and data collection improves. Obviously, for each new condition studied, new vignettes would need to be produced, but we believe that this method is versatile and can be replicated for other conditions of interest.

### Compliance with instructions

Some technical issues arose in the evaluation of the study. Participants were originally requested to have six blank records created for the study. Piloting found this to be overly onerous and participants commented that they were reluctant to request so many records be created, so the instruction was reduced to one record. One participant documented in a previously used “dummy” record. Even this was interpretable by acknowledging, then ignoring, previously recorded data that was visible on the first vignette’s screenshot.

Telephone support was only required by one participant, and this was for help downloading the video. Another participant also could not view the video did not seek help but completed the study using audio alone. Both of these issues were due to outdated software on participants’ computers.

The participant who reported a code they had entered was not visible on their screenshot shows an awareness of the study aims and limitations and is suspected to have been an EHR specific issue (this EHR software has since been retired). Participants were instructed to flag this issue in the instruction video, and the fact of this issue being reported is regarded as evidence of the success of this mode of instruction.

Participants were asked not to prescribe or write management plans for the vignettes as this would have been a test of knowledge and would also have required more of their time. Those that did, may have done so due to the realism of the scenario, or because they did not fully read or listen to the instructions. The two participants that completed the questionnaire electronically rather than by printing it on paper caused difficulty in analysing the Visual Analogue Scales but this has no implication for vignette interpretation.

The poor return rates of summary screens and even lower return rates of blank referral letters restrict comments on the EHRs’ automatic code transfer and requiring these elements of the study may need to be abandoned although, again, the bulk of the study remains unaffected by this and may indicate that too much was being requested from the participants.

### Strengths and limitations

As noted previously, other methods for eliciting clinician-recording practices result in uncontrollable variables, particularly in terms of the patient presentation. The strength of this new method is that it standardizes many of the variables at the patient end of the interaction, thus allowing us to examine in isolation the variation in the codes GPs use in response to an identical stimulus. It is reproducible and can be conducted economically on a large scale with no additional investment, as it forgoes the expense of complex camera rigs and actors and the time needed for coordination of these activities.

One limitation is that vignettes are less natural than real or simulated two-way consultations and limit the potential range of entries. Participants’ written requests for more clinical detail and the paucity of agreement (53%) with the statement “There was enough information to record the consultation effectively” relating to all vignettes, indicate the inevitable artificiality of a simulated patient monologue. These vignettes were designed to be slightly ambiguous in order to reflect the diagnostic uncertainty experienced in primary care, but participants expressed a desire for more information on test results and answers to rule in/rule out questions. Precisely what additional information they wanted varied between doctors. In the future, some vignettes could be included that have more information and clearer clinical presentations, enabling us to explore how these changes impact on coding and documentation.

A balance is required between including too much or too little information in the vignettes to ensure participants’ feel comfortable assigning a Read code, but also to keep the time of the study short enough to ensure uptake. Participants may have interpreted “does this vignette reflect real-life?” as a comment on the process rather than the particular content. Similar uncertainty exists around participants’ interpretation of “quality” and whether the rating referred to clinical, technical, research or acting quality. Nonetheless, both were generally rated highly, and the fidelity is considered adequate for the method’s aims. It is clear each monologue must be carefully constructed in order to generate useful results and these questions could be clarified in future studies.

The method, in its current form, is limited to what is included in the questionnaire when interpreting reasons behind data entry. Responses allowed some exploration of rationale behind the assignment of particular codes, but post-participation elicitation interviews, as sometimes used in video studies, would further our understanding of actions and the decision making processes.

### Future refinements

In future work refining this method, unintended variables such as poor quality digital compression of JPEG images into video formats should be minimized by ensuring high levels of quality in the presentation of images within vignettes. Test results or letters from secondary care could be incorporated into vignettes to see how they impact documentation practices.

Minor technical challenges, such as being unable to watch or download video, can be overcome by using up-to-date remote questionnaire software, such as Qualtrics (29), which allows video embedding and participant file upload. Another refinement would be the use of a technology such as VPN to enable clinicians to access remotely an environment in which all relevant software is available, with dummy records ready for completion. These changes, if allowed and compatible with NHS hardware, would avoid many of the technical issues that we encountered. Further improvements could include a short video of a clinician completing the study to accompany the written instructions. Whilst this could enhance understanding of, and compliance with, study instructions, it would probably not negate the need for a researcher to be on-call for technical support.

The amount of time required to complete the study was, in some cases, double that predicted and this is likely to have been a factor in the low return rate of the non-core elements (blank referral letters and summary screens) of data collected. Thus we suggest limiting future studies to four vignettes to keep the process achievable in clinical practice. Increasing the incentive to participate beyond a raffle prize draw may improve recruitment levels and the completion of all tasks requested. Ideally, clinicians should be remunerated for the time invested in the study.

Screenshot analysis requires a degree of awareness of which descriptors are codes, as they may not be immediately identifiable. Additionally, it is possible that not all relevant data will be captured on the screenshot. Employing an attending researcher to explore a subsample of the EHR entry directly, or target screenshots that suggest unusual or missing data, would enable us to estimate the prevalence of such errors.

### Clinical Implications

A recent NICE publication (30), on the diagnosis and management of drug allergy, highlights how poor clinical documentation is a major issue, with the inability of the EHR to distinguish between intolerance and allergy, leading to incorrect labelling of patients and an adverse impact on their care. Allergy specifically warrants further investigation and this technique may be very useful in doing so. Understanding how data are recorded and used in general practice was introduced as a core competency in the 2016 RCGP training curriculum (31). This includes being aware of how to contribute patient data to, and the value of, large GP databases to facilitate epidemiological and drug safety research as well as service planning. This novel method could be used by GPs as a professional training tool in documentation techniques and to explain the importance and significance of code assignment.

### Implications for research

EHR market shares fluctuate and, in response, some repositories such as CPRD are acquiring new data sources to maintain data levels (32). Those that do so may encounter unpredictable compatibility issues due to differences in EHR software and Read code version. This technique allows identification of any differences in recording practices between clinicians using different EHRs and could better enable integration. It could also be used as an adjunct to code-based research studies in order to identify how clinicians document (including a likely range of codes) when encountering various presentations of the condition of interest. Although this study focussed on the now retired Read code system, it is equally applicable to other clinical coding systems including SNOMED, OPCS and ICD (33, 34).

A further area of interest in EHR research is the use of free text and the balance between it and codes. Free text is currently not extracted for privacy reasons and so clinical information recorded in text is lost to research. This technique could be used to explore the quantity and quality of information that may be lost to free text when only coded data is extracted from EHRs.

Finally, although this study focussed on the use of primary care EHRs, it is a versatile technique and would find equally valid application in the rapidly developing fields of secondary care EHRs.

## Conclusion

To the best of the authors’ knowledge, filmed monologue vignettes have not previously been used to explore documentation within the EHR. This novel, reproducible, method enabled standardization of multiple variables that affect the study of EHR use including history taking, communication skills and doctor-patient interactions. Based on quantitative and qualitative feedback it is considered a viable, resource-light method for gaining accurate, new insights into the field of EHR documentation. Clinicians used their usual EHR, in their own environment, at their convenience and required minimal supervisory input, providing benefits to both researcher and participant. The method has significant potential for all EHR stakeholders and could be used as part of a training intervention, or in research, to identify the range of codes used by practitioners. We anticipate this method may have a particular impact on the planning and interpretation of EHR-based research studies.

## Supplementary Files

Supplementary File 1 Participant pack including questionnaire

Supplementary File 2 Transcript of user instructions
