# Supplementary material for: Filmed Monologue Vignettes: a novel method for investigating how clinicians document consultations in electronic health records
Source: Int J Popul Data Sci. 2018 Nov 14;3(1):430. doi: 10.23889/ijpds.v3i1.430 (PMC8142957; doi:10.23889/ijpds.v3i1.430)
Supplement: Supplementary File 1 Participant pack including questionnaire [file ijpds-03-430-s001.pdf]

brighton and sussex medical school

Mayfield House

University of Brighton

Falmer

BN1 9PH

<Researcher email address>

<Researcher telephone number>

<Date>

Dear Practice Manager,

Re: Allergic presentations in primary care
R&D Ref No: 13/085/SMI

A GP trainee +/- their trainer (the participants) from your practice have kindly agreed to participate in the above research study. This study forms the basis of a Master's degree dissertation into the documentation of allergy within primary care and their participation is very much appreciated. It is hoped that the results of the study will help to improve patient safety. **It is neither a knowledge test nor will the results reflect upon the practice in any way nor does it involve real patients.**

The participants will be sent a USB memory stick including videos of acted patient consultations. The files will have been checked for viruses by the university antivirus software (SOPHOS); however it is advised that local virus check software is also used.

The participants have been asked to document the consultations and will require a blank "dummy" patient record to be created for them. The name of this "dummy" patient need only be told to the study participant(s) so they can access them. We would be extremely grateful if you could ensure this vital task is performed to enable their participation. Please do not tell the participant's each other's dummy patient name.

Unfortunately, we do not have any financial reimbursement available for engagement in the study. All participants have generously offered their own time to assist. We hope that you and your team will also be able to offer your time to help with this important research.

Should you have any queries, please do not hesitate to contact us.

Yours sincerely,

Professor Helen Smith and Dr Simon Glew

Mayfield House

University of Brighton

Falmer

BN1 9PH

<Researcher email address>

<Researcher telephone number>

<Date>

Dear Participant,

Re: Allergic presentations in primary care

R&D Ref No: 13/085/SMI

Thank you for agreeing to participate in this study. Please find enclosed a USB memory stick along with all the information you will need to complete the study. Although we have scanned the files with the university antivirus software (SOPHOS) it is advised that you use a local virus checker before accessing them. Please play the file named "introduction" and have the paper questionnaire to hand to complete as you watch each vignette. You should be able to complete the research by saving all documents to the USB stick and hand writing on the questionnaire. Please refer to the step-by-step guided on the included paper document to assist with this if necessary. If you are unable to save anything to the USB stick please print the relevant output.

Remember to plan to complete the study **independent** of your trainer/trainee. Please return all data, including the USB stick, in the stamped addressed envelope provided as soon as possible.

Should you have any questions or difficulties please contact Dr Glew at your convenience.

Yours sincerely,

Professor Helen Smith and Dr Simon Glew

Allergic presentations in primary care: Vignette Questionnaire and overall feedback

Please answer these few questions on the vignettes after having watched each one. They have been designed to require as little of your time as possible. Remember, if it is difficult to see where your input has been recorded on any of your screen prints please write where it is located on the screen i.e. "top left corner of screen" next to the relevant vignette below.

Please contact Simon Glew if you have any questions: <email address> or <telephone number>.

How to print screen:

Note:
Some keyboards or mobile PCs that don't have the PRINT SCREEN button might use other keyboard combinations, such as FN+INSERT, to take a screen capture. Check the information that came with your computer or the manufacturer's website for more information.

Windows 8: Press the Windows logo key +PrtScn (PRTSC or PRTSCN on some keyboards & usually located around the top right of the keyboard). The screen will dim for a moment when the screen is copied and saved as a file in the Screenshots folder (which is in your Pictures library). Please then transfer this file to the USB stick. Name it according to the appropriate vignette number.

http://windows.microsoft.com/en-us/windows/take-screen-capture-print-screen#take-screen-capture-print-screen=windows-8

Windows 7: Press the PrtScn key (PRTSC or PRTSCN on some keyboards & usually located around the top right of the keyboard). When you press it, an image of your screen is copied to the Clipboard. This is called a screen capture. To save the screen capture, paste it into Paint or another image-editing program and save it as a file on the USB stick. Name it according to the appropriate vignette number.

http://windows.microsoft.com/en-us/windows/take-screen-capture-print-screen#take-screen-capture-print-screen=windows-7

Windows Vista: Press PRINT SCREEN (PRTSC or PRTSCN on some keyboards & usually located around the top right of the keyboard). There are two types of screen captures you can take: the entire screen, or just the active window. For example, if you have three programs open at the same time, you might want a screen capture of only the active window.

To copy the entire screen
Press PRINT SCREEN.
To copy only an active window
Press ALT+PRINT SCREEN.
To print the screen capture, paste it into Paint or another image-editing program and save it as a file on the USB stick. Name it according to the appropriate vignette number.
http://windows.microsoft.com/en-us/windows/take-screen-capture-print-screen#take-screen-capture-print-screen=windows-vista

Vignette 1:

Were you able to assign a Read code you were satisfied with? Yes/No

If No: why not?

Read code unavailable Unable to find a suitable Read code

Multiple Read codes are suitable Other (please describe):

Do you think the free text is important in this scenario?

Place an "X" on the line in the appropriate position.

Extremely important Not at all important

Please explain your response

Do you feel you had enough information to record the consultation effectively? Yes/No

If No: what more would you like to know?

Vignette 2:

Were you able to assign a Read code you were satisfied with? Yes/No

If No: why not?

Read code unavailable Unable to find a suitable Read code

Multiple Read codes are suitable Other (please describe):

Do you think the free text is important in this scenario?

Place an "X" on the line in the appropriate position.

Extremely important Not at all important

Please explain your response

Do you feel you had enough information to record the consultation effectively? Yes/No

If No: what more would you like to know?

Vignette 3:

Were you able to assign a Read code you were satisfied with? Yes/No

If No: why not?

Read code unavailable Unable to find a suitable Read code

Multiple Read codes are suitable Other (please describe):

Do you think the free text is important in this scenario?

Place an "X" on the line in the appropriate position.

Extremely important Not at all important

Please explain your response

Do you feel you had enough information to record the consultation effectively? Yes/No

If No: what more would you like to know?

Vignette 4:

Were you able to assign a Read code you were satisfied with? Yes/No

If No: why not?

Read code unavailable Unable to find a suitable Read code

Multiple Read codes are suitable Other (please describe):

Do you think the free text is important in this scenario?

Place an "X" on the line in the appropriate position.

Extremely important Not at all important

Please explain your response

Do you feel you had enough information to record the consultation effectively? Yes/No

If No: what more would you like to know?

Vignette 5:

Were you able to assign a Read code you were satisfied with? Yes/No

If No: why not?

Read code unavailable Unable to find a suitable Read code

Multiple Read codes are suitable Other (please describe):

Do you think the free text is important in this scenario?

Place an "X" on the line in the appropriate position.

Extremely important Not at all important

Please explain your response

Do you feel you had enough information to record the consultation effectively? Yes/No

If No: what more would you like to know?

Vignette 6:

Were you able to assign a Read code you were satisfied with? Yes/No

If No: why not?

Read code unavailable — Unable to find a suitable Read code

Multiple Read codes are suitable — Other (please describe):

Do you think the free text is important in this scenario?

Place an "X" on the line in the appropriate position.

Extremely important — Not at all important

Please explain your response

Do you feel you had enough information to record the consultation effectively? Yes/No

If No: what more would you like to know?

Personal information:

Age:

Gender:

Stage of training or years since qualified as a GP:

Trainees only: Please describe any other experience in addition to Foundation training and VTS.

Previous experience of allergy:

None/1-5 cases/5-10 cases/10-20 cases/20-40 cases/>40 cases

How long have you worked in this GP practice?

What computer system do you use? (i.e. Vision/Systemone/EMIS)

What version is it?

How long have you used this computer system for?

How confident do you feel that you are using this computer system appropriately?

Place an "X" on the line in the appropriate position.

Extremely Confident Extremely uncertain

Have you ever used any other GP computer systems?

If yes: which and for how long?

In real-life practice, how often are you able to assign the Read code you want in order to accurately code a consultation?

Place an "X" on the line in the appropriate position.

Always Never

In real-life practice, do you feel you have a tendency to use the same repertoire of codes that you are familiar with and have used previously?

Do you feel the following affect how you code a consultation (please ring where appropriate):

Prescribing decision Financial incentives Your familiarity with a condition

Previous codes used within the patient's record Other:

The overall process:

This is the first time a study like this has been conducted. Would you be willing to be contacted by telephone (5 minutes or so) to discuss it further?

Yes/No

If yes: Please write your number and a when would be most convenient to contact you.

Please rate the quality of the vignettes and whether you feel they reflected real life scenarios:

Vignette	Quality of the vignette out of 10 (1 being poor, 5 being average, 10 being excellent)	Did this vignette reflect a real life scenario (Y or N)?
1. Unwell after eating prawns		
2. Neck rash		
3. Penicillin hospital admission		
4.Simvastatin aches		
5. Autumn cough and wheeze		
6.Egg vomit and rash		

Were there any problems understanding the instructions? (Please contact Simon Glew as soon as possible if these have prevented you completing the study)

Did any problems arise with the use of the files or the computer (compatibility etc)? Yes/No

If yes: Please describe in more detail

What did you find most challenging about the task?

Has participating changed your interest in primary care research?

Not at all Decreased it Increased it

Any comments/suggestions:

Thank you for participating in this study!
