# Supplementary material for: Filmed Monologue Vignettes: a novel method for investigating how clinicians document consultations in electronic health records
Source: Int J Popul Data Sci. 2018 Nov 14;3(1):430. doi: 10.23889/ijpds.v3i1.430 (PMC8142957; doi:10.23889/ijpds.v3i1.430)
Supplement: Supplementary File 2 Transcript of user instructions [file ijpds-03-430-s002.pdf]

Dissertation Film script and Gold standard Read codes

Introduction:

Hello and thank you for agreeing to participate in this research which forms the basis of my Masters dissertation.

This study aims to review GPs documentation of common allergic presentations to primary care.

What you need to do:

Please ensure that you are completing this study alone and independent of your trainer or trainee or any other participants. Please do not discuss the study with one another until after you have all completed and returned your answers to me.

Please open the blank patient record that has been created especially for this project. Your practice manager will have kindly arranged for this and made you aware of the “dummy” patient name. In a moment you will be shown a series of vignettes. Please watch each vignette in its entirety before entering any information into the record. Document each consultation as if the patients are presenting to you in your surgery. Please capture the history and diagnosis to the level of detail you would in reality. Don’t worry about creating a management plan or prescribing anything: this is not the focus of the study.

Once you have done that please take a screenshot of the patient record. If you are unsure how to do this, please read the file “how to print screen” or contact me. Ensure that the study data you have input is clearly visible and as large as possible but also ensure that any data unrelated to the study is hidden. Open the “paint” application on your desktop and paste the print screen image into this, either by pressing “ctrl” + “v” at the same time or “paste” via the “paint” taskbar. If you are unable to find the paint program you could alternatively paste the screen print into Word, although the quality is worse and harder to analyse.

Please save this file to a suitable location as vignette 1,2,3 or 4 etc. If you feel there may be difficulty in identifying where the data you have input is on the screen print; please print the page and highlight this or make a note on the questionnaire next to the relevant vignette for instance “under vignette 3: write allergy input top left corner of screen”. If you get stuck at all with the process, again, please read the file “how to print screen” or contact me.

If you are working in a practice where you can produce your own letters for referrals, please also create a standard medical referral letter for each vignette. You do not have to type anything into the letter; this is to review what information is transferred automatically in onward communications that may or may not relate to the patient’s presentation. For example, if the patient were referred to secondary care in future. Please save this as an appropriately named file (letter vignette 1,2,3,4 etc) in a convenient place or, if this isn’t possible, please print it.

Once you have done this please complete the paper questionnaire for the relevant vignette and then move on to the next one. There are six short vignettes in total. Please complete the questionnaire for each vignette as you progress rather than after watching all the vignettes. After completing vignette six there is an important final part of the questionnaire to complete.

Please email or upload the files as soon as possible and return any print outs and the questionnaire in the stamped addressed envelope provided. If you requested a memory stick with the electronic files on please save the output files to it and return that in the envelope along with any paper output data.

If you have any problems or questions please contact me as soon as possible. Thank you once again for taking part.

Vignette scripts

Vignette 1: Food allergy

15 year old girl:

"I've been feeling really unwell lately after eating prawns. After about half an hour they make my stomach hurt; it really cramps and I throw up a couple of times and have some diarrhoea. If I think about it, I also get really itchy skin and my lips start to tingle. I've never had anything like it happen before this year and now it's happened a few times."

NOT TO BE READ OUT : READ CODE: SN530 Allergic reaction OR SN53. Allergy, unspecified OR SN58. Food allergy OR SM80. Fish and shellfish causing toxic effect OR SM80X Toxic effect of other fish and shellfish poisoning SyuGA [X]Toxic effect of other fish and shellfish poisoning

Vignette 2: Nickel allergy

22 year old lady, wearing a necklace:

I've got this rash on the back of my neck doctor. It's been really itchy, in fact that's how I first noticed it, I got my partner to have a look and they said I had a rash there. It's started to develop these blisters; *<show photo of contact nickel rash>*, do you think it's an insect bite?

What, this? *<holds up necklace>* Oh I've worn this for years. My gran gave it to me before she died.

NOT TO BE READ OUT: READ CODE: M128. Allergic contact dermatitis OR SM5y3 Nickel compounds causing toxic effect or M12y811 Nickel sensitivity

Vignette 3: Antibiotic allergy

A concerned mother:

Hello doctor, I don't think we've met before? My family and I are new to the area and I wanted to talk to you about my son, Billy. I'm sure you've got his record up there on your screen because that's who the appointment is for but he's in school today. He's six now but when he was four, at our old surgery, he was given penicillin for a sore throat. Within half an hour I had to call an ambulance. His breathing went really noisy and he started to look extremely unwell. It was really scary. He had to go to hospital within half an hour as he was having a severe anaphylactic reaction. He had steroids and adrenaline in hospital and was an inpatient for 3 days. I just want to make sure that it's on his record so it doesn't happen

again. Oh, no, I don't need you to prescribe anything today; I'll ring you another time to sort that.

NOT TO BE READ OUT: There are two elements to code here; anaphylaxis and the penicillin allergy *14L.. SN501 Anaphy shock due/adv efect/correct drug or med properly admin. 14M5. H/O: anaphylactic shock SN50. Anaphylactic shock H/O: drug allergy OR TJ0.. Adverse reaction to antibiotics OR TJ00. Adverse reaction to penicillins OR TJ00z Adverse reaction to penicillin NOS*

Vignette 4: Medicine intolerance

58 year old man

Oh doctor, thanks for seeing me. I've been feeling awful these last 2 weeks. Ever since you started me on that tablet for my cholesterol, whatsit? Simvastatin. I've been having these aches in my arms and my legs, they feel really tender as well and I've lost some strength as well. I'm sure it's those tablets y'know?

NOT TO BE READ OUT: READ CODE: TJC24 Adverse reaction to simvastatin

Vignette 5: Aeroallergen

20 year old male student

It happens around the same time every year doctor: Always in the Autumn; between September and December really. I get a cough and a bit wheezey and I can't seem to stop my nose running. The rest of the year I'm absolutely fine and I compete in triathalons. I've never needed to see a doctor before.

NOT TO BE READ OUT: READ CODE: H171. Allergic rhinitis due to other allergens OR Hyu20 [X]Other seasonal allergic rhinitis

Vignette 6: Egg allergy

A father regarding his 9 month-old child.

Hello doctor. I'm really worried about my little boy Charlie. He's 9 months old now as you know and really he's been absolutely fine up until now apart from a bit of baby eczema. He's been growing really well and he's more advanced than the other babies at nursery his age.

It was several weeks ago now that there was an episode where I gave him some scrambled egg. He went really quiet and I knew something was wrong and then he was violently sick.

I wondered if it was a one off, so I gave him a little bit of egg the other day and red splodges came up on his face. I took a photo of it on my phone; here <*show photo of urticarial rash*>. He loves eating cakes and biscuits and they have no effect, but I am steering clear of anything too eggy, like scrambled eggs, now.

NOT TO BE READ OUT: READ CODE: SN580 Egg allergy OR SN581 Egg protein allergy
